# Does phosphorylation of cofilin affect the progression of human bladder cancer?

**DOI:** 10.1186/1471-2407-13-45

**Published:** 2013-02-01

**Authors:** Hong Chung, Bokyung Kim, Seung-Hyo Jung, Kyung-Jong Won, Xiaowen Jiang, Chang-Kwon Lee, So Dug Lim, Sang-Kuk Yang, Ki Hak Song, Hong Sup Kim

**Affiliations:** 1 Departments of Urology, School of Medicine, Konkuk University, 82 Gugwon-daero, Chungju, Chungbuk, 380-704, Republic of Korea; 2 Department of Physiology, School of Medicine, Konkuk University, 82 Gugwon-daero, Chungju, Chungbuk, 380-704, Republic of Korea; 3 Department of Pathology, School of Medicine, Konkuk University, Hwayang-dong, Gwanjin-gu, Seoul, 143-792, Republic of Korea; 4 Departments of Urology, College of Medicine, Chungnam National University, Chungnam, Republic of Korea

**Keywords:** Cofilin, Phosphorylation, Invasion, Urothelial cell carcinoma

## Abstract

**Background:**

We determined the differently expressed protein profiles and their functions in bladder cancer tissues with the aim of identifying possible target proteins and underlying molecular mechanisms for taking part in their progression.

**Methods:**

We examined the expression of proteins by proteomic analysis and western blot in normal urothelium, non-muscle-invasive bladder cancers (NMIBCs), and muscle-invasive bladder cancers (MIBCs). The function of cofilin was analyzed using T24 human bladder cancer cells.

**Results:**

The expression levels of 12 proteins were altered between bladder cancers and normal bladder tissues. Of these proteins, 14-3-3σ was upregulated in both NMIBCs and MIBCs compared with controls. On the other hand, myosin regulatory light chain 2, galectin**-**1**,** lipid-binding AI, annexin V, transthyretin, CARD-inhibitor of NF-κB-activating ligand, and actin prepeptide were downregulated in cancer samples. Cofilin, an actin-depolymerizing factor, was prominent in both NMIBCs and MIBCs compared with normal bladder tissues. Furthermore, we confirmed that cofilin phosphorylation was more prominent in MIBCs than in NMIBCs using immunoblotting and immunohistochemcal analyses. Epidermal growth factor (EGF) increased the phosphorylation of cofilin and elevated the migration in T24 cells. Knockdown of cofilin expression with small interfering RNA attenuated the T24 cell migration in response to EGF.

**Conclusions:**

These results demonstrate that the increased expression and phosphorylation of cofilin might play a role in the occurrence and invasiveness of bladder cancer. We suspected that changes in cofilin expression may participate in the progression of the bladder cancer.

## Background

Bladder cancer is the ninth most often diagnosed and the seventh most prevalent cancer worldwide, and shows an increasing tendency in Asia [1]. It commonly presents as an urothelial cell carcinoma with non-muscle-invasive bladder cancer (NMIBC), but is clinically well controlled and can be treated relatively easily by transurethral resection of bladder tumor. However, bladder cancer has a high recurrence rate at 60–70%, and 11% of the recurrent cases progress to a muscle-invasive bladder cancer (MIBC) [2,3]. It is very difficult to predict recurrence or progression, or to understand bladder carcinogenesis according to established clinical classification system. Moreover, there are no available markers that can guide clinicians in diagnosis, recurrence, or in decreasing the number of unnecessary cystoscopy among patients with bladder cancer. Therefore, new pathological and biological markers for the recurrence and progression of bladder cancers are needed.

There are several known markers used clinically for bladder cancer: nuclear matrix protein 22, telomerase, epidermal growth factor (EGF) receptor and others [4-8]. However, these have limitations in their specificity and/or sensitivity, as is shown by cystoscopy of bladder cancers [4]. Celis’ group has performed proteomic and genomic analyses to identify markers in bladder cancers [9-12]. Differentially expressed proteins, such as adseverin, profiling 1, ADAM28, and annexin 1, have been identified as markers for bladder cancer [13-16]. Moreover, real-time polymerase chain reaction has been used to detect RNA related with muscle invasive bladder cancer [17]. Proteomic analysis has also been performed using urinary and plasma proteins from patients with bladder cancers [18,19]. The mechanisms for bladder cancer development and progression have not yet been fully resolved and as such the need for more reliable and accurate biomarkers for disease recurrence and progression remains. Protein modifications such as phosphorylation, glycosylation or oxidation play vital roles in the initiation and progression of many molecular pathways. Hence, an understanding of protein modification is crucial for identifying the key functional modulators of carcinogenesis, progression and metastasis [20]. Although previous studies have investigated human bladder cancer tissues using proteomic tools [13-16], these mainly involved protein identification and immunohistochemistry. Therefore, further studies are required to characterize the mechanisms of progression and invasion and to explore for potential targets for occurring mechanisms of MIBC and NMIBC.

The aims of the present study were to identify proteins that are involved in bladder cancer progression by comparing protein expression patterns between normal urothelium tissues, NMIBCs, and MIBCs samples using proteomic technique and to determine the underlying molecular mechanism associated with the observed protein changes. We found an actin-depolymerizing factor (ADF), cofilin, to be elevated in NMIBC and MIBC tissues and further confirmed its function in cell motility using T24 human bladder cancer cells.

## Methods

### Materials

A total of 24 bladder samples that used in this study were obtained from patients with bladder cancers and bladder rupture (Table 1; n = 6, normal urothelium; 9, NMIBCs; 9, MIBCs). Tumors were graded according to WHO criteria and staged according to the TNM classification. All of the materials used for two-dimensional electrophoresis (2-DE) and mass spectrometry (MS) were purchased from BioRad (Hercules, CA, USA) or Applied Biosystems (Foster City, CA, USA). Recombinant human EGF was purchased from R&D Systems (Minneapolis, MN, USA). McCoy’s 5A medium was obtained from Welgene (Daegu, Korea). Polyclonal anti-phosphorylated ser-3 cofilin and anti-cofilin antibodies were obtained from Cell Signaling (Beverly, MA, USA). Polyclonal anti-GAPDH antibody and chemical reagents were purchased from Sigma-Aldrich (St. Louis, MO, USA).

**Table 1:** Clinical, histological and epidemiological characteristics of the patients whose bladder proteins were analyzed

**Sex**	**Age**	**Clinical diagnosis**	**Cytology**	**Pathologic diagnosis**	**Tumor grade**	**TNM stage**
Normal urothelium, Bladder						
M	32	Traumatic bladder rupture	Class I	Normal Urothelium		
F	35	Traumatic bladder rupture	Class I	Normal Urothelium		
M	30	Traumatic bladder rupture	Class I	Normal Urothelium		
F	44	Traumatic bladder rupture	Class I	Normal Urothelium		
F	38	Traumatic bladder rupture	Class I	Normal Urothelium		
F	41	Traumatic bladder rupture	Class I	Normal Urothelium		
Non-muscle-invasive urothelial cell cancer, Bladder						
M	67	Bladder tumor	Class I	Urothelial cell carcinoma	Grade 1	TaN0M0
M	71	Bladder tumor	Class II	Urothelial cell carcinoma	Grade 2	T1N0M0
M	69	Bladder tumor	Class II	Urothelial cell carcinoma	Grade 1	TaN0M0
F	66	Bladder tumor	Class II	Urothelial cell carcinoma	Grade 1	TaN0M0
F	62	Bladder tumor	Class II	Urothelial cell carcinoma	Grade 1	T1N0M0
M	69	Bladder tumor	Class I	Urothelial cell carcinoma	Grade 2	T1N0M0
M	53	Bladder tumor	Class I	Urothelial cell carcinoma	Grade 1	T1N0M0
F	68	Bladder tumor	Class I	Urothelial cell carcinoma	Grade 1	TaN0M0
M	69	Bladder tumor	Class II	Urothelial cell carcinoma	Grade 1	TaN0M0
Muscle-invasive urothelial cell cancer, Bladder						
M	68	Bladder tumor	Class III	Urothelial cell carcinoma	Grade 3	T4aN1M0
F	72	Bladder tumor	Class IV	Urothelial cell carcinoma	Grade 3	T4aNxM0
M	63	Bladder tumor	Class IV	Urothelial cell carcinoma	Grade 3	T4bN1Mx
M	67	Bladder tumor	Class IV	Urothelial cell carcinoma	Grade 3	T4bN2M0
F	71	Bladder tumor	Class III	Urothelial cell carcinoma	Grade 3	T4bN2Mx
M	69	Bladder tumor	Class IV	Urothelial cell carcinoma	Grade 2	T4bN2M0
M	63	Bladder tumor	Class IV	Urothelial cell carcinoma	Grade 3	T4aNxM0
M	68	Bladder tumor	Class V	Urothelial cell carcinoma	Grade 3	T4aN1M0
M	53	Bladder tumor	Class IV	Urothelial cell carcinoma	Grade 3	T4aNxM0

### Preparation of bladder tissue samples

All experiments were performed in accordance with the institutional guidelines of Konkuk University and were approved by the Institutional Review Board of Konkuk University Chungju Hospital, Chungju-city, Korea (KUCH 07-009). All patients had given their informed consent to participate in the study. Urothelial samples (0.1−1 mg) from bladder rupture were obtained from the patients with intraperitoneal bladder rupture. The abdomen was opened through a vertical lower mid-line incision and then the ruptured margin of the bladder was incised using Metzenbaum scissors. NMIBC and MIBC tissues were removed by bladder biopsy or transurethral resection. After removal of the tissues, these were rinsed in physiological salt solution (in mM; NaCl 136.9, KCl 5.4, CaCl_2_ 1.5, MgCl_2_ 1.0, NaHCO_3_ 23.8, EDTA 0.01). The samples were then snap-frozen in liquid N_2_ for proteomic and western blot analyses. The samples for immunohistochemical assay were immersed in 4% paraformaldehyde for 8 hr and embedded in paraffin wax.

### 2-DE and MALDI-TOF/TOF MS

Bladder tissue samples from bladder rupture and cancer patients were homogenized in 2-DE buffer containing 8 M urea, 2 M thiourea, 100 mM DTT, 4% CHAPS and 1 × complete protease inhibitor cocktail (Roche Applied Science, Penzberg, Germany). The homogenates were incubated for 40 min and then centrifuged at 12 000 × *g* for 10 min at 10°C. The supernatants were diluted with rehydration buffer containing 7 M urea, 2 M thiourea, 100 mM DTT, 2% CHAPS, 0.5% ampolyte and 0.01% bromophenol blue, and then used for 2-DE as described in our previous report [21-23]. Images of silver-stained gel were visualized using a densitometer (VersoDoc Imaging System 1000; Bio-Rad). The gels obtained from six independent experiments were normalized as a percentage of the total spot volume in all of the spots present on the gels and analyzed statistically using PDQuest software (Version 7.1.1, Bio-Rad).

In-gel digestion and protein identification were performed as reported [21-23]. Briefly, the protein spots were digested with trypsin and desalted with ZipTip C_18_ (Millipore, Bedford, MA, USA). The peptide samples were mixed with CHCA matrix solution and then analyzed by MALDI-TOF/TOF (AB4700, Applied Biosystems) in the reflector mode. The search parameters were used trypsin, 2 missed cleavage, cut-off individual ion scores > 20, extensive homology *p* < 0.05, variable modification of carbamidomethyl, oxidation, propionamide and pyro-glu (*N*-term), a peptide charge of 1+, and monoisotopic. The mass accuracy was within 100 ppm for the mass measurement and within 0.2 Da for CID experiments. Spectra were processed and analyzed with Global Protein Server Explorer 3.0 software (Applied Biosystems). The internal Mascot program (Matrix Science Ltd., London, UK) was used for matching MS and MS/MS data against database information. The resulting data were surveyed against human databases downloaded from NCBI and the Swiss Prot/TrEMBL homepages.

### Immunoblotting

For immunoblotting, extracted protein samples were diluted 1:1 (v/v) with SDS sample buffer (40 mM Tris–HCl pH 6.8, 8 mM EGTA, 4% 2-mercaptoethanol, 40% glycerol, 0.01% bromophenol blue, and 4% SDS) and then denatured by boiling for 5 min. The samples (20–30 μg/lane) were separated by 14% SDS-PAGE and transferred electrophoretically onto polyvinylidene fluoride membranes (Millipore, Bedford, MA, USA). The membrane was blocked for 2 hr with phosphate-buffered saline containing 0.05% Tween 20 and 5% fat-free dried milk. The membrane was incubated overnight with antibodies diluted 1:1,000 and then reacted with horseradish peroxidase-conjugated antibodies (Amersham Pharmacia Biotech Inc., Piscataway, NJ, USA) for 1 hr. The blots were visualized with enhanced chemiluminescence reagents (Amersham Pharmacia). Statistical analysis was performed using Quantitation software (Bio-Rad).

### Immunohistochemistry

Total 24 bladder tissues from each patients were used for the immunohistochemical analysis. Each formalin-fixed and paraffin-embedded tissue was cut into 4-mm thick sections, deparaffinized, rehydrated and blocked with methanol containing 3% hydrogen peroxide. Cofilin antibodies diluted 1:100 was then applied and incubated for 60 min in a room temperature. After washing, the sections were incubated with horseradish peroxidase-conjugated dextran polymer reagent kits (ChemMate Envision Kit K5007; DakoCytomation, Glostrup, Denmark) for 30 min. Peroxidase activity was visualized with 3, 3’-diaminobenzidine tetrachloride according to the manufacturer’s instructions. The sections were counterstained with hematoxylin at room temperature. Negative controls were carried out by omitting the primary antibodies. The cytoplasmic and nuclear expression of both antibodies was semiquantatively scored in three groups as follows: diffuse strong staining (> 50%), weak or focal staining (< 50%), and absence of any staining.

### Human bladder carcinoma cell line and transfection

Human bladder cancer T24 cells that were purchased from American Type Culture Collection (ATCC, Manassas, VA, USA) were cultured in McCoy’s 5A medium containing 10% FBS, 100 U/mL penicillin, 100 μg/mL streptomycin, and 200 mM glutamine. The cultured cells (8 × 10^4^) were replaced with FBS-free McCoy’s 5A medium, and then transfected with the siRNA or nonsilencing control RNA to 1,000 pM using a transfection reagent (Welfect-QTM Gold, Welgene, Daegu, Korea). The relative protein expression levels of cofilin were examined using immunoblotting analysis with anti-cofilin antibody. Cofilin siRNA was designed to target the human cofilin sequence 5'-CCCAAACUGCUU UUGAUCU-3' (Accession number: NM_005507; Bioneer, Daejeon, Korea). Control nonsilencing RNA was purchased from Bioneer.

### Migration assay

Migration assays were performed in 48-well microchemotaxis Boyden chambers (Neuro Probe, Cabin John, MD, USA). Polycarbonate membranes (8-μm pore size, Neuro Probe) were coated with a 0.1 mg/mL of type I collagen (BD Bioscience, San Diego, CA, USA) and then dried for 60 min. Cells were harvested using trypsin-EDTA (Life Technologies, Paisley, UK) and resuspended in McCoy’s 5A medium containing 0.1% BSA with or without EGF. The bottom chamber was loaded with 3 × 10^4^ cells and the membrane was laid over the cells. The microchamber was then inverted and incubated at 37°C for 80 min. The membranes were fixed and stained using Diff-Quik (Baxter Healthcare, Miami, FL, USA). The number of cells migrated through the membrane was determined by counting four randomly chosen regions of each well under a microscope (× 400).

### Statistical analysis

Data are presented as the mean ± SD. The statistical evaluation of data was performed using Student’s t-tests for comparisons between pairs of groups and ANOVA for multiple comparisons; *p* < 0.05 was considered to be a statistically significant difference.

## Results

### Isolation and identification of differentially expressed proteins between normal and bladder cancer tissues

**Figure 1:**
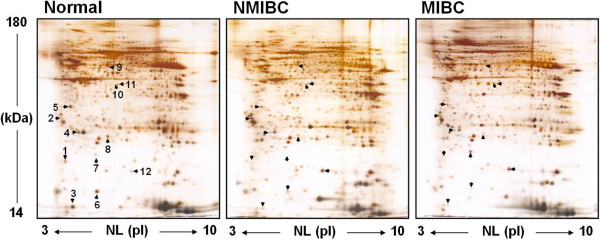
**2-DE gel images showing protein expression in bladder tissues from normal human, NMIBC and MIBC samples.** The protein samples were loaded onto nonlinear IPG strips (pH 3-10, 17 cm) in an IEF cell and then separated by 12% SDS-PAGE. The protein spots were visualized by silver staining. The numbers are the spot numbers of detected proteins and arrows indicate the differentially expressed proteins in bladder cancer tissues compared with controls. Representative images from six independent experiments.

**Figure 2:**
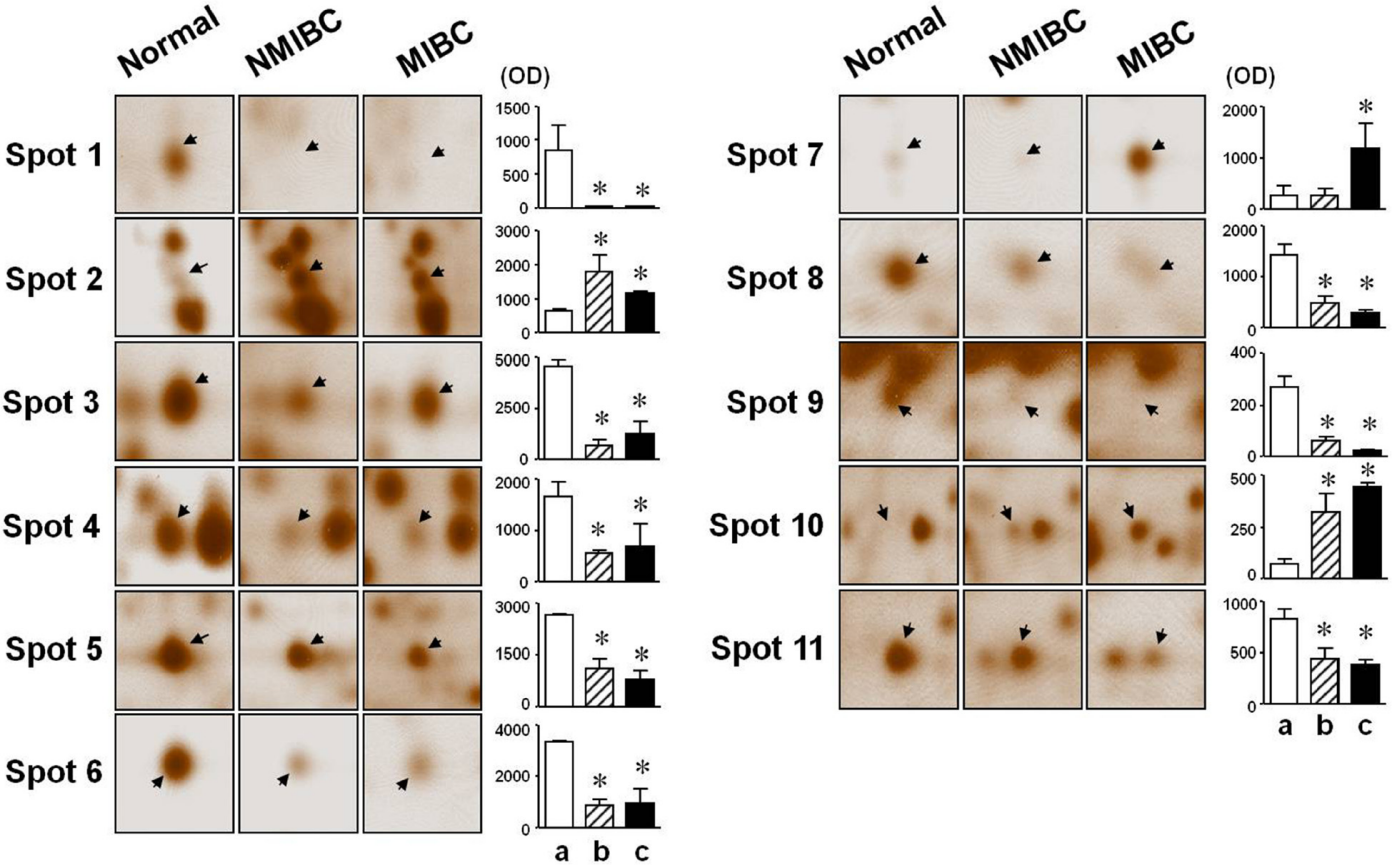
**Expression profiles and quantitative analyses of up or downregulated proteins in NMIBC, and MIBC tissues compared with controls.** Arrows on the cropped 2-DE-gels represent proteins spots showing statistically significant changes between cancer groups and controls. Data were obtained from 2-DE gels of six independent experiments using PDQuest software;* *p* < 0.05 (n = 6). White bars (**a**) indicate normal human bladder tissues; striped bar (**b**) show bladder tissue from patients with NMIBC; black bars (**c**) indicate MIBC samples.

First, we analyzed the differences in protein expression levels between normal and bladder cancer tissues. The mean matching rates for gels were about 67–72% for the same cancer developmental stage and 60–67% between gels for different developmental stages. Figure 1 shows the expression pattern of proteins in normal bladder, NMIBC and MIBC tissues. The expression level of 12 protein spots was altered by at least 1.5-fold in bladder tissues from cancer patients compared with those obtained from controls (Figure 2). The differentially expressed proteins were identified by MALDI-TOF/TOF mass spectrometry (Table 2). Of these proteins, 14-3-3σ (spot 2), macrophage-capping protein (spot 10), and cofilin (spot 12) were upregulated in both NMIBC and MIBC samples compared with normal human bladder tissues. On the other hand, myosin regulatory light chain 2 (spot 1**),** galectin-1 (spot 3), lipid-binding AI (spot 4), annexin V (spot 5), transthyretin (spot 6), CARD-inhibitor of NF-κB-activating ligand (spot 8), actin prepeptide (spot 9), and macrophage-capping protein (spot 11) were downregulated in bladder tissues from NMIBC and MIBC samples compared with controls (Figure 2). Ferritin light subunit (spot 7) was only upregulated in the cancer tissues from patients with MBIC compared with the normal bladder tissues. In contrast, there was no difference in the expression level of ferritin light subunit between normal bladder and NMIBC tissues. Table 2 shows the characteristics of identified protein spots including representative peptide sequences, sequence coverage, theoretical and experimental pI and Mr values, accession numbers from both the Swiss-Prot and NCBI databases, and known functions of the identified proteins.

**Table 2:** Identification of differentially expressed proteins in bladder tissue from normal human, non-muscle invasive and muscle-invasive bladder cancer patients

**No**	**Change folds**		**Protein names**	**Peptide sequences**	**Score/SC**^**1)**^**(%)**	**pI/Mr (kDa) theoretical (experimental)**	**Accession No/database**	**Known function**
	**Non-in**^**1)**^**/normal**	**Invas**^**1)**^**/normal**						
1	−31.16	−64.91	Myosin regulatory light chain 2	GNFNYVEFTR	32/5	4.80/19.7	P24844/SP	Regulation of cell contractile activity
						(4.75/21.1)		
2	2.75	1.78	14-3-3 protein σ	EMPPTNPIR	20/3	4.72/27.8	631131/NC	Epithelial cell growth
						(4.68/30.0)	P31947/SP	
3	−3.83	−3.65	Galectin-1	DSNNLCLHFNPR	141/16	5.34/14.7	30582389/NC	Regulation of apoptosis, proliferation and differentiation
				DGGAWGTEQR		(4.80/15.3)	P09382/SP	
4	−2.92	−2.39	Lipid-binding AI	DEPPQSPWDR	75/8	5.27/28.3	229513/NC	Lipid binding protein
				THLAPYSDELR		(4.88/25.8)		
5	−2.38	−3.31	Annexin V	GTVTDFPGFDER	160/11	4.94/35.8	809189/NC	Anticoagulant
				LYDAYELK				
				FITIFGTR		(4.78/34.1)	P08758/SP	
				SEIDLFNIR				
6	−3.78	−3.50	Transthyretin	GSPAINVAVHVFR	196/41	5.33/12.8	339685/NC	Transports of thyroxine
				AADDTWEPFASGK				
				ALGISPFHEHAEVVFTANDSGPR		(5.07/17.0)	P02766/SP	
7	1	4.56	Ferritin light	KPAEDEWGKTPDAMK	130/32	5.65/16.3	182516/NC	Iron homeostasis
			Subunit	KLNQALLDLHALGSAR		(5.06/22.3)	P02792/SP	
				LGGPEAGLGEYLFER				
8	−2.97	−4.88	CARD-inhibitor of	DPYPVSYLR	37/2	5.14/48.8	15617462/NC	Inhibitor of NF-κB activation
			NF-κB-activating			(5.18/25.3)		
			Ligand					
9	−4.44	−12.69	Actin prepeptide	AGFAGDDAPR	85/11	5.19/36.8	178067/NC	Cell motility
				AVFPSIVGRPR		(5.19/52.8)	P62736/SP	
				SYELPDGQVITIGNER				
10	4.53	5.71	Macrophage capping protein	EVQGNESDLFMSYFPR	76/8	5.32/38.5	21730367/NC	Regulation of cytoplasmic and/or nuclear structures
				QAALQVAEGFISR		(5.40/39.6)	P40121/SP	
11	−1.86	−2.31	Macrophage capping protein	EVQGNESDLFMSYFPR	117/12	5.32/38.5	21730367/NC	Regulation of cytoplasmic and/or nuclear structures
				QAALQVAEGFISR		(5.70/39.6)	P40121/SP	
				MQYAPNTQVEILPQGR				
12	4.25	4.41	Cofilin	AVLFCLSEDKK	98/21	8.26/18.4	P23528/SP	Actin polymerization
				YALYDATYETK		(7.48/19.8)		
				HELQANCYEEVKDR				

^**1)**^non-in, non-muscle-invasive bladder cancer; invas, muscle-invasive bladder cancer; SC, sequencer coverage.

### Changes in cofilin level in cancer tissues from patients with cancer

**Figure 3:**
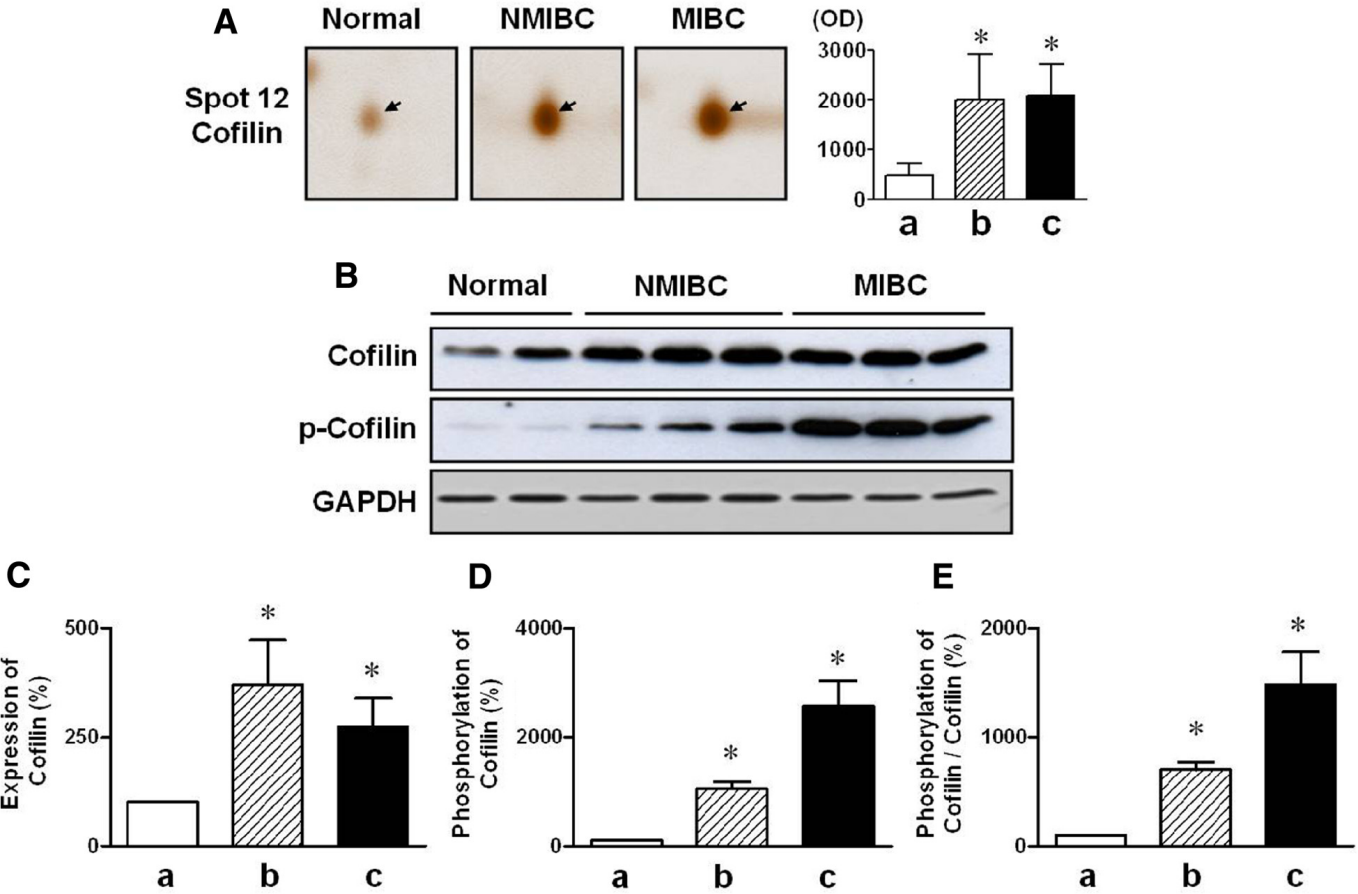
**Expression and phosphorylation levels of cofilin in bladder tissues isolated from NMIBC, and MIBC tissues.** (**A**) Enlargement of cofilin spots (arrows) from 2-DE gel images obtained from normal bladder tissue, NMIBC and MIBC samples. (**B**) Western blot analysis of proteins extracted from normal human bladder, NMIBC and MIBC samples. (CE) The statistical results obtained from panel B. Expression (**C**), phosphorylation (**D**) and ratio (**E**) of phosphorylated to total cofilin. * *p* < 0.05. White bars (**a**), normal bladder tissue (control); striped bar (**b**), NMIBC tissue; black bars (**c**), MIBC tissue.

**Figure 4:**
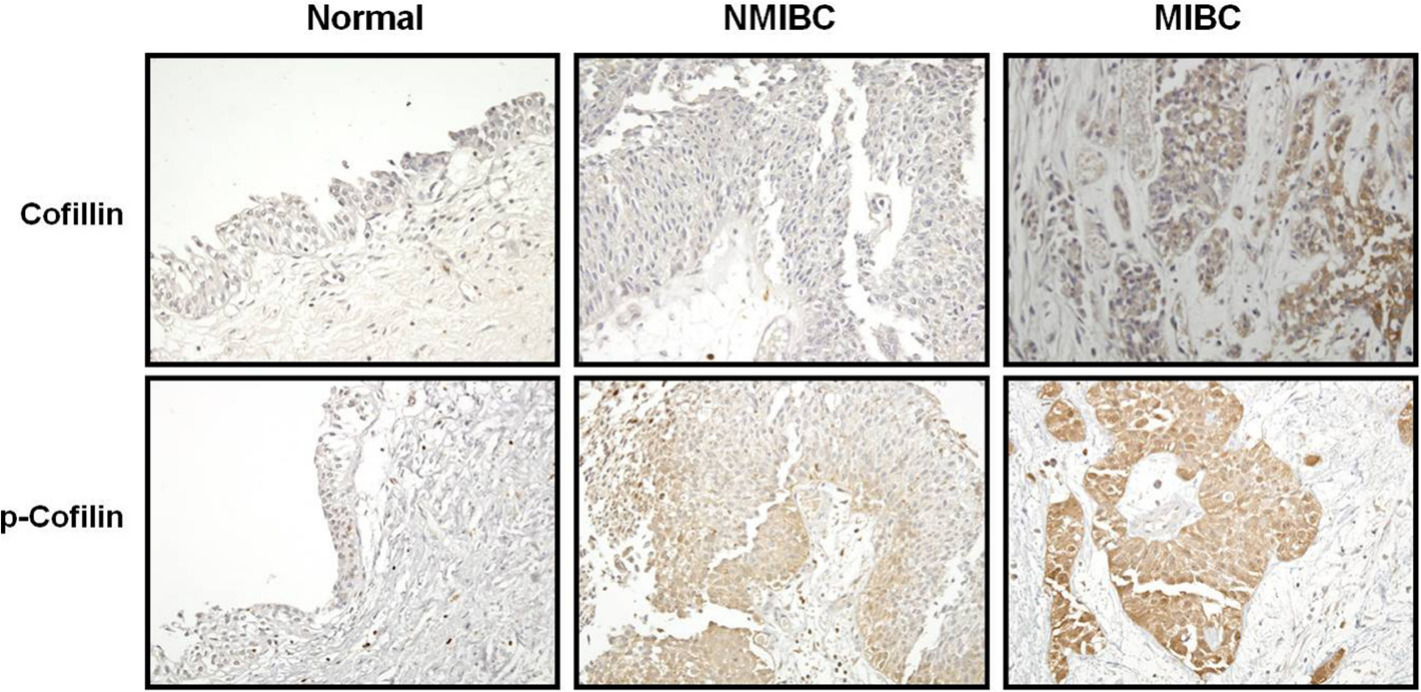
**Immunohistochemical analysis of cofilin and phosphorylated cofilin in bladder tissues from bladder rupture and from patients with noninvasive and invasive cancers.** Paraffin-wax-embedded sections were processed using polyclonal antibodies to cofilin and phosphorylated cofilin. A negative control was performed by omitting the primary antibodies.

As shown in Figure 3A, the expressed level of cofilin was increased markedly in both NMIBC and MIBC tissues compared with normal bladder tissues. To confirm the results of 2-DE and silver staining analysis, we examined the expression level of cofilin in bladder tissues from controls and patients with bladder cancers using immunoblotting and immunohistochemical analyses. Immunoblotting showed that cofilin expression was elevated in both NMIBC and MIBC samples compared with the normal bladder tissues (Figure 3B). There were statistical significant differences in the expression and phosphorylation of cofilin and ratio of phosphorylated cofilin/total cofilin in bladder cancer tissue compared with normal human bladder tissue (Figure 3C-E). The function of cofilin is tightly regulated by its phosphorylation and dephosphorylation levels [24,25], and implicated in the cancer cell motility and metastasis [26-28]. Therefore, we focused our analysis on the phosphorylation level of cofilin in tissues from normal human bladder and patients with bladder cancers. The phosphorylation of cofilin was elevated in both NMIBC and MIBC samples compared with the normal bladder tissues, and was more prominent in MIBCs than in NMIBCs (Figures 3, 4).

By immunohistochemistry, antibodies against cofilin and phosphorylated cofilin revealed a negative immunoreactivity in the normal bladder tissues. Cofilin stained diffusely and strongly in all MIBCs and NMIBCs, whereas phosphorylated cofilin stained more in all MIBCs compared with NMIBCs (Figure 4).

### Role of cofilin in the motility of T24 human bladder cancer cells

**Figure 5:**
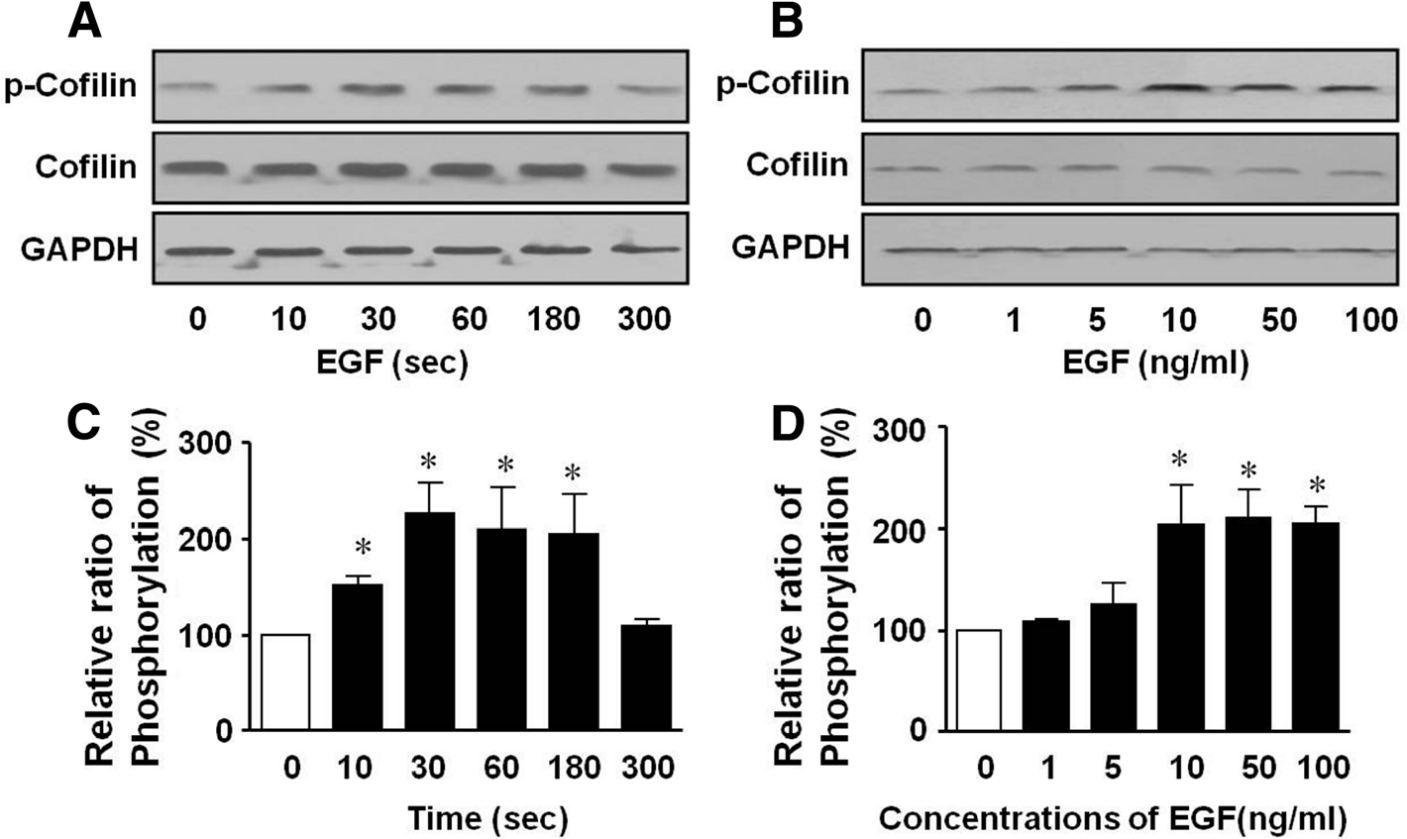
**EGF-induced phosphorylation of cofilin in human bladder cancer T24 cells were stimulated with EGF (50 ng/mL) for the indicated times (A), and with a range of EGF concentrations (1–100 ng/mL) for 30 sec (B).** The cell lysates were immunoblotted with anti-phosphorylated ser-3 and anti-nonphosphorylated cofilin antibodies. (**C**) and (**D**) show statistical results obtained from the upper panels (**A**) and (**B**), respectively. The basal levels of cofilin phosphorylation are expressed as 100%. * denotes a statistically significantly difference from the basal levels of cofilin phosphorylation (*p* < 0.05).

Recently it was reported that cofilin is implicated in cell migration in various cells such as smooth muscle cells and metastatic cancer cells [26,27] and EGF induces this cell behavior [28,29]. Therefore, the role of cofilin in migration was determined using T24 human bladder cancer cells. Cofilin phosphorylation that was induced in response to 50 ng/mL EGF showed a maximal response at 30 sec (227.3 ± 30.0% of the control, Figure 5A and 5C), then gradually dropped over 10 min and reached a quiescent level. As shown in Figure 5B and 5D, cofilin phosphorylation was dose dependently increased by EGF treatment (1-100 ng/mL), and showed a maximal response at 50 ng/mL (210.1 ± 27.8% of control). However, the EGF treatment did not influence the levels of cofilin or GAPDH expression.

**Figure 6:**
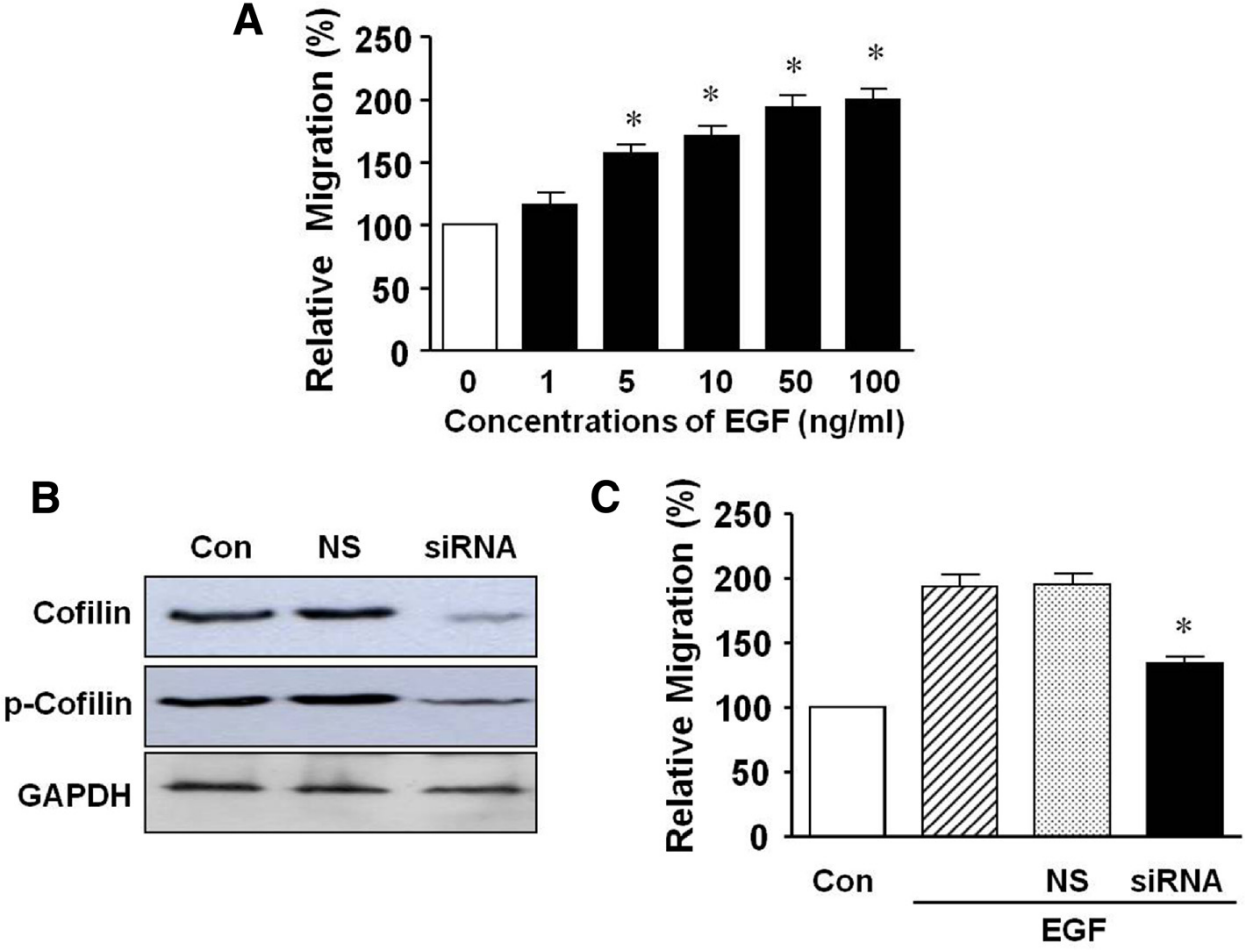
**Effects of cofilin knockdown on EGF-induced migration in human bladder cancer T24 cells.** (**A**) The effect of EGF on cell migration. Cells were treated with EGF (1–100 ng/mL) for 80 min and migration was quantified with a Boyden microchemotaxis chamber assay. (**B**) Non-phosphorylated and phosphorylated cofilin expression in human bladder cancer T24 cells transfected with small interfering (si)RNA for cofilin. (**C**) Effects of siRNA-cofilin transfection on EGF-induced migration. The transfected cells were treated with EGF (50 ng/mL) and then subjected to migration assays. Migration in the quiescent state is expressed as 100%. * *p* < 0.05. Con, control; NS, nonsilencing siRNA; siRNA, siRNA for cofilin; p-cofilin, phosphorylated cofilin.

We also tested the induction of migration in response to EGF in T24 human bladder cancer cells. As shown in Figure 6A, EGF (1-100 ng/mL) increased migration of T24 cells in a dose-dependent manner, peaking at 100 ng/mL. To determine the role of cofilin phosphorylation in bladder cancer, we tested the effects of cofilin knockdown on EGF-induced migration of T24 human bladder cancer cells. The expression and phosphorylation levels of cofilin were decreased dramatically in cells that were transfected with siRNA-cofilin (Figure 6B). Moreover, siRNA-cofilin significantly inhibited cell migration (134.6 ± 3.24% of control) in response to EGF (50 ng/mL) (Figure 6C). The responses in cells transfected with nonsilencing RNA was not significantly different from the non-transfected controls.

## Discussion

In proteomics analysis, we found that the expression of cofilin was significantly increased in bladder cancers without difference between NMIBC and MIBC samples. This was confirmed by immunoblotting with an anti-cofilin antibody. Cofilin is found in bladder cancer cell lines [30]. It is a ubiquitously expressed ADF in a variety of cells and plays a crucial role in the formation of actin filaments by regulating polymerization and depolymerization [29]. Hence, cofilin is capable of stimulating the disassembly and severing of actin filaments at or near the pointed end, thereby continuously supplying actin for polymerizing and rapid turnover of actin filaments [27,29]. The actin cytoskeleton is an essential framework for the control of a variety of cellular functions and is required in cell migration. Cofilin is the most abundant isoform of ADF found in invasive tumor cells [31]. Up and downregulation of cofilin correlate with increases and decreases, respectively, in the motility of tumor cells [32]. Cofilin is also associated with carcinoma progression and is a marker for breast cancer [33,34]. Reactive oxygen species (ROS) are involved in the development of cancer [35], and cofilin expression was changed during high ROS states in vascular smooth muscle [22]. Cofilin is dynamically regulated by cycles of phosphorylation such that the local concentrations of kinases and phosphatases determine the overall balance of cofilin activity [36]. Thereafter, we suspected that changes in cofilin expression are involved in the activation of cell-cycle and consequently participate in the progression of the bladder cancer. Presently, there are no clinically acceptable markers for early initial diagnosis or diagnosis of bladder cancer recurrences and guide us in reducing the frequency of the need for cystoscopy in patients with bladder cancer. Therefore, new biological and prognostic markers for the prediction of tumor recurrence and progression are needed. Further analysis using urine or blood samples will clarify the possibility of cofilin as an available biomarker of MIBC or NMIBC.

The activity of cofilin is regulated by phosphorylation of its ser-3 residue, which is induced by LIM kinase (LIMK) 1 and 2, or related testicular protein kinase (TESK) types 1 and 2 [26]. Several phosphatases, such as slingshot and chronophin, control the activity of cofilin via dephosphorylation [27,30] and are tightly associated with the invasion of cancer cells [34]. These results imply that cofilin phosphorylation has a role in determining the invasion and metastasis of cancer cells. In our results, the cofilin expression increased in both NMIBC and MIBC compared to normal bladder tissues. However, there were no significant differences in NMIBC and MIBC. In addition, we found that phosphorylated cofilin was greater in MIBC than in NMIBC. Moreover, the ratio of phosphorylated to total cofilin was also higher in MIBC than in NMIBC (Figure 3). Taken together, we suggest that the increased expression and phosphorylation of cofilin might be involved in the occurrence and invasiveness of bladder cancer, respectively. Thereafter, our findings indicate that cofilin could be a therapeutic target in preventing the occurrence and invasiveness of bladder cancers.

In this study, EGF elevated the phosphorylation of cofilin without altering its expression level and induced the migration of T24 bladder cancer cells. These responses were inhibited in bladder cancer cells when cofilin expression was blocked with siRNA-cofilin, confirming that cofilin participates in the motility of bladder cancer cells. EGF induces the invasion and metastasis of cancer cells [37] and the EGF receptor is over-expressed in a number of human malignancies such as cancers of the lung, brain, breast and bladder [38,39]. EGF also increases the activity of LIMK in carcinoma cells and LIMK is upregulated in invasive mammary carcinomas [31,33]. These results imply that cofilin phosphorylation participates in the motile response to EGF in bladder cancer cells. Therefore, it appears that EGF is involved in bladder cancer cell invasion via cofilin phosphorylation.

Two protein spots corresponding with macrophage-capping protein were observed in the whole proteome from both human bladder cancers and normal tissues. Moreover, the acidic form of macrophage-capping protein (spot 10) was increased significantly, but the basic form (spot 11) was decreased in bladder cancer tissues compared with controls, without any change in total expression. Modifications involving changes in molecular weight and/or pI observed on 2-DE gels are implicated in the activation of proteins in a variety of cells [23]. Previously, we reported that changes in phosphorylation and pI shift of proteins were found during oxidative stress in vascular smooth muscle and hypertensive vessels [22]. These results imply that the modification, but not total expression, of macrophage-capping protein occurs during bladder cancer progression. Although macrophage-capping protein expression was found to be increased in leukemic cancer cells [40], the physiological and pathophysiological roles of this protein have not been fully determined. Further analysis will establish the potential of cofilin as a biomarker for MIBC or NMIBC.

Previous reports demonstrate that annexin V and transthyretin were downregulated and galectin-1 was increased in bladder cancer [41-43]. Moreover, we identified lipid-binding AI, ferritin light subunit, and CARD-inhibitor of NF-κB that have not been reported in bladder tissues. In this study, bladder cancers showed upregulated expression of 14-3-3σ (spot 2) whereas myosin regulatory light chain 2 (spot 1), galectin-1 (spot 3), lipid-binding AI (spot 4), annexin V (spot 5), transthyretin (spot 6), CARD-inhibitor of NF-κB-activating ligand (spot 8) and actin prepeptide (spot 9) were downregulated. Some of these changed proteins, as well as cofilin are related to cell motility. Myosin regulatory light chain 2 and 14-3-3σ are known as proteins that regulate cell motility, including migration, invasion and metastasis. Moreover, 14-3-3ζ binds to cofilin and increases its phosphorylation by inhibiting cofilin phosphatase [44]. From these results, it can be assumed that the 14-3-3 protein participates in the progression of bladder cancer via cofilin phosphorylation. In contrast to the present result, a previous report showed that 14-3-3 σ was downregulated in MIBC tissues [9]. This discrepancy could be explained by a difference in the isoforms expressed on 2-DE gels. Here, we also found that the expression of ferritin light subunit (spot 7) was greater, those of CARD-inhibitor of NF-κB-activating ligand (spot 8) and actin prepeptide (spot 9) lesser in MIBC than in NMIBC tissues. Therefore, beside cofilin phosphorylation, ferritin light subunit, CARD-inhibitor of NF-κB-activating ligand and actin prepeptide could be a marker of invasive cancers compared with NMIBC.

## Conclusions

We found that the expression levels of 12 proteins were altered in bladder cancers compared with normal bladder tissue. Of these changes in cofilin expression levels were prominent in both NMIBC and MIBC. Cofilin phosphorylation was greater in MIBC than in NMIBC. Knockdown of cofilin attenuated the EGF-induced migrations of T24 human bladder cancer cells. Our results showed that the increases in the expression and phosphorylation of cofilin might play a crucial role in the occurrence and invasiveness of bladder cancers. It is suggested that cofilin phosphorylation could participate to the invasiveness of human bladder cancer.

## Competing interests

The authors declare that they have no competing interests.

## Authors’ contributions

HC participated in study design and drafted the manuscript. BK participated in coordination and helped to draft the manuscript. S-HJ and C-KL performed 2-DE, MALDI-TOF/TOF, and immunoblot analysis. K-JW and XJ carried out culture of cell line and transfection. SDL carried out immunohistochemistry. S-KY assisted data collection. KHS performed statistical analysis and helped to draft the manuscript. HSK participated in study design and coordination, helped to draft the manuscript. All authors read and approved the final manuscript.

## Pre-publication history

The pre-publication history for this paper can be accessed here:

http://www.biomedcentral.com/1471-2407/13/45/prepub
